# MALT1 Protease Activity Is Required for Innate and Adaptive Immune Responses

**DOI:** 10.1371/journal.pone.0127083

**Published:** 2015-05-12

**Authors:** Jong W. Yu, Sandy Hoffman, Allison M. Beal, Angela Dykon, Michael A. Ringenberg, Anna C. Hughes, Lauren Dare, Amber D. Anderson, Joshua Finger, Viera Kasparcova, David Rickard, Scott B. Berger, Joshi Ramanjulu, John G. Emery, Peter J. Gough, John Bertin, Kevin P. Foley

**Affiliations:** 1 Pattern Recognition Receptor Discovery Performance Unit, Immuno-Inflammation Therapeutic Area, GlaxoSmithKline, Collegeville, United States of America; 2 Department of Safety Assessment, GlaxoSmithKline, King of Prussia, United States of America; 3 Quantitative Sciences, GlaxoSmithKline, Collegeville, United States of America; Heinrich-Heine-University and University Hospital Duesseldorf, GERMANY

## Abstract

CARMA-BCL10-MALT1 signalosomes play important roles in antigen receptor signaling and other pathways. Previous studies have suggested that as part of this complex, MALT1 functions as both a scaffolding protein to activate NF-κB through recruitment of ubiquitin ligases, and as a protease to cleave and inactivate downstream inhibitory signaling proteins. However, our understanding of the relative importance of these two distinct MALT1 activities has been hampered by a lack of selective MALT1 protease inhibitors with suitable pharmacologic properties. To fully investigate the role of MALT1 protease activity, we generated mice homozygous for a protease-dead mutation in MALT1. We found that some, but not all, MALT1 functions in immune cells were dependent upon its protease activity. Protease-dead mice had defects in the generation of splenic marginal zone and peritoneal B1 B cells. CD4^+^ and CD8^+^ T cells displayed decreased T cell receptor-stimulated proliferation and IL-2 production while B cell receptor-stimulated proliferation was partially dependent on protease activity. In dendritic cells, stimulation of cytokine production through the Dectin-1, Dectin-2, and Mincle C-type lectin receptors was also found to be partially dependent upon protease activity. *In vivo*, protease-dead mice had reduced basal immunoglobulin levels, and showed defective responses to immunization with T-dependent and T-independent antigens. Surprisingly, despite these decreased responses, MALT1 protease-dead mice, but not MALT1 null mice, developed mixed inflammatory cell infiltrates in multiple organs, suggesting MALT1 protease activity plays a role in immune homeostasis. These findings highlight the importance of MALT1 protease activity in multiple immune cell types, and in integrating immune responses *in vivo*.

## Introduction

The discovery that the *MALT1* (Entrez Gene ID 10892) and *BCL10* genes are involved in translocations commonly found in B-cell lymphomas of mucosa-associated lymphoid tissue (MALT lymphomas) in turn led to the identification of the so-called ‘CBM signalosome’, which is a heterotrimeric complex between any one of several different members of the CARMA protein family, with BCL10 and MALT1 [1]. CBM complexes have emerged as important regulators of NF-κB-mediated signaling, not only in tumorigenesis, but also in normal physiology downstream of many different receptors [1]. For example, the CARMA1-containing CBM complex has been implicated in signaling through the T cell receptor (TCR) [2–4], and a CBM complex containing the CARMA-related protein CARD9 mediates signaling downstream of the Dectin-1 and Dectin-2 C-type lectin receptors (CLRs) [5–7].

In T and B lymphocytes, antigen receptor engagement results in stimulation of the canonical NF-κB signaling pathway. This is due in part to Protein Kinase C-mediated phosphorylation of CARMA1 and its assembly into a CBM signalosome [1,8,9]. As part of the CBM complex, oligomerized MALT1 then acts as a scaffold to recruit critical downstream signaling proteins, such as the ubiquitin ligase TRAF6 which enables K63-polyubiquitination of the regulatory subunit of IKK, NEMO, leading to phosphorylation of IκB [10–13]. The ensuing proteosomal degradation of IκB permits NF-κB nuclear translocation and transcription of genes involved in lymphocyte proliferation, differentiation, and effector functions.

The importance of the CBM signalosome in antigen receptor signaling has been demonstrated using mice in which the *Malt1* gene was knocked out [14,15]. In these studies, MALT1 was found to be required for TCR/CD28-costimulation of T cell proliferation and IL-2 production through activation of NF-κB. However, MALT1 is not required for positive or negative selection of thymocytes [16], and its role in BCR signaling remains controversial [14,15,17]. MALT1 knockout mice also displayed defects in the development of marginal zone (MZ) B cells in the spleen and peritoneal B1 B cells [14,15]. Further, basal serum immunoglobulin levels were severely reduced, and the *in vivo* response to T-dependent and T-independent antigens was compromised [14,15].

MALT1 has also been implicated in receptor-mediated signal transduction in a wide variety of other cell types [1]. In dendritic cells, a CARD9-containing CBM complex activates NF-κB downstream of the C-type lectin receptor (CLR) Dectin-1 [6,7,18]. Two other CLRs, Dectin-2 and Mincle, have also been shown to signal through CARD9 [19], suggestive of a role for MALT1 in initiation of innate anti-microbial responses and directing adaptive immune responses through production of cytokines that promote T helper cell differentiation to the Th1 and Th17 subsets [7,18,19]. MALT1 has also been implicated in NF-κB activation in mast cells following FcεRI activation [20] and in fibroblasts following lysophorphatidic acid treatment [21], as well as in MAPK activation following NK cell receptor stimulation [22].

MALT1 contains a domain that is related to the protease active sites of caspases [23], and the role of MALT1 protease activity has been investigated using an irreversible protease inhibitor, Z-VRPR-FMK. These studies are challenging to interpret due to the potential off-target activity and poor pharmacologic properties of this compound, which necessitate the use of very high concentrations in cellular assays, as well as preventing its use *in vivo*. However, studies using Z-VRPR-FMK have suggested that MALT1 protease activity plays an important role in immune cells. For example, MALT1 protease activity is thought to contribute to optimal TCR responses [24–27], but is not required for TCR-induced IκB phosphorylation and degradation, suggesting a modulatory role independent of IKK activation [25,28–30]. In support of this view, MALT1 proteolysis of the inhibitory deubiquitinases A20 and CYLD potentiates NF-κB and JNK, respectively [24,31], while cleavage of the RNase Regnase-1 prolongs mRNA stability of T cell effector genes [30]. In addition, proteolysis of RelB is proposed to release suppression of RelA and c-Rel to enhance NF-κB activation independent of the IKK complex [32]. MALT1 can also cleave its signaling partner BCL10 [25]. These multiple layers of signaling inhibition regulated by MALT1 protease activity suggest that this function is pivotal in executing antigen receptor responses.

Given the importance of MALT1 in lymphocyte activation and adaptive immune responses, and its emerging role in innate immunity [6,20,22], it is of central importance to unravel the relative contributions of MALT1 scaffolding and protease activities. To this end, we describe the immune properties of homozygous MALT1 protease-dead knockin (*Malt1* ^PD/PD^) mice in comparison to homozygous MALT1 null knockout (*Malt1* ^-/-^) mice. *Ex vivo*, we found that many key cellular functions are driven by MALT1 protease activity, including T cell activation and CLR-induced production of some cytokines by dendritic cells. However, a distinct set of MALT1-dependent functions are independent of MALT1 protease activity, and presumably represent functions that only require MALT1 scaffolding activity. *In vivo*, MALT1 protease activity was essential for the development of innate-like B cell populations and the maintenance of basal immunoglobulin levels. Further, Ig-responses to immunization with both T-dependent and T-independent antigens were dependent upon MALT1 protease activity. However, despite the generally decreased responses observed *ex vivo* and *in vivo* in *Malt1* ^PD/PD^ mice, these animals also developed inflammatory cell infiltrates in multiple organs. These results demonstrate that MALT1 protease activity plays key roles in both innate and adaptive immune responses, and in regulating immune homeostasis *in vivo*.

## Materials and Methods

### Mice

**Fig 1:**
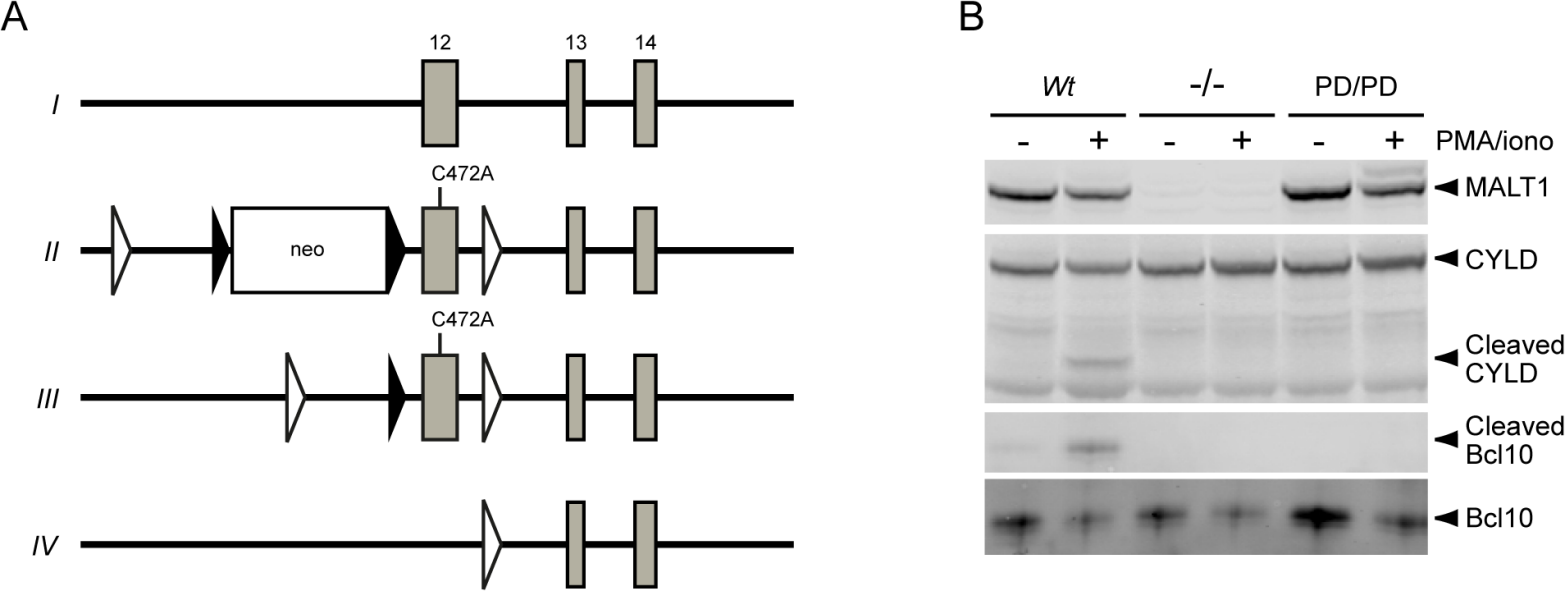
A C472A mutation in MALT1 inactivates protease activity without changing protein expression. (A) Homologous recombination of the *Wt Malt1* locus (*I*) in C57BL/6-derived embryonic stem cells was used to introduce a catalytically inactive C472A mutation into exon 12 of the *Malt1* gene (for clarity, the genomic locus is not draw to scale). The resulting neo cassette-containing mice (*II*) were crossed to a Flp recombinase-expressing deleter mouse strain to generate animals carrying the protease-dead allele (*III*) used in these studies. These mice were in turn crossed to a Cre recombinase-expressing deleter strain to excise exon 12 and generate a null allele (*IV*). Close triangles, FRT sites; open triangles, *loxP* sites. (B) Purified total B cells from the spleens of *Wt*, *Malt1* ^-/-^, and *Malt1* ^PD/PD^ mice were treated with (+) or without (-) PMA plus ionomycin for 1 h and assessed by Western blotting for expression of MALT1, CYLD, Bcl10, and proteolytically cleaved CYLD and Bcl10. For clarity, the images were cropped. Uncropped western blots are shown in S1 Fig (Complete western blots confirming MALT1 (C472A) expression and loss of protease activity).

*Malt1* ^PD/PD^ and *Malt1* ^-/-^ lines were generated by genOway (Lyon, France) using animals and breeding facilities at Charles River Laboratories (Lyon, France). The mouse *Malt1* locus (with the start and stop codons in exons 1 and 18, respectively) was modified by homologous recombination in C57BL/6-derived embryonic stem cells in order to introduce a C472A (TGT>GCC; numbering based on Uniprot Q2TBA3-1) mutation in exon 12 (Fig 1A). Mutation of this active site catalytic cysteine has previously been shown to inactivate MALT1 protease activity [24,25]. *Malt1* ^PD/PD^ mice lacking the neo cassette, and *Malt1* ^-/-^ mice lacking exon 12 [which results in a premature stop codon in exon 13; since the endogenous poly(A) site is preserved in the truncated mRNA and there are 5 splice junctions between the stop codon and the poly(A), this strategy was predicted to induce nonsense-mediated mRNA decay and result in a null allele], were generated by breeding to C57BL/6-Flp and C57BL/6-Cre deleter lines, respectively. *Malt1* ^PD/PD^ mice were genotyped by PCR using primers 5’-CAGAGAAAGACAAACACGTGGTCAG-3’and 5’-CAATATCATTTTGGGCTATTGAGGTAG-3’, yielding 289 bp and 385 bp bands for the wild type (*Wt*) and C472A alleles, respectively. *Malt1* ^-/-^ mice were genotyped by PCR using primers 5’-GGATGCTAAGGCAGGAGGATT-3’ and 5’-ACCAGTTCATACTTGGCTTCTTT-3’, yielding 975 bp and 205 bp bands for the *Wt* and null alleles, respectively. For each line, heterozygous mice were interbred and progeny were born at the expected Mendalian ratio. Real-time RT-PCR of splenic total RNA confirmed that the C472A allele did not alter *Malt1* gene expression in heterozygous *Malt1* ^+/PD^ or *Malt1* ^PD/PD^ mice, and the presence of the mutation was confirmed by sequencing. *Malt1* ^PD/PD^ and *Malt1* ^-/-^ mice appeared outwardly normal at weaning, although *Malt1* ^PD/PD^ mice did displayed an ~10% decrease in body weight relative to *Wt* mice at 8 weeks of age. *Malt1* ^PD/PD^ mice were maintained under specific pathogen-free conditions in sterilized microisolator cages, and studies were performed between 7–15 weeks of age.

### Ethics statement

All animal studies conducted at genOway or Charles River Laboratories were ethically reviewed and approved by each institution’s Institutional Animal Care and Use Committee and were carried out in accordance with European Directive 2010/63/EU and the GlaxoSmithKline (GSK) Policy on the Care, Welfare and Treatment of Animals. All studies conducted at GSK were conducted in accordance with the latter policy and were approved by the GSK Institutional Animal Care and Use Committee. Euthanasia was performed by CO_2_ asphyxiation followed by either cervical dislocation or thoracotomy; every effort was made to minimize suffering.

### Immunoblotting

B cells were purified from splenocytes using mouse CD45R (B220) MicroBeads (Miltenyi Biotec), and subsequently stimulated with 200 ng/ml phorbol 12-myristate 13-acetate (Sigma) and 1 μM ionomycin (EMD Millipore) for 1 h at 37°C. For S2 Fig (RelB cleavage is impaired in activated B cells expressing MALT1 (C472A)), all samples were also treated with 10 μM MG-132 (Sigma, #M7449-1ML) at the time of stimulation. Cells were lysed in RIPA buffer containing a protease inhibitor cocktail (Roche), subjected to SDS-PAGE, and transferred to nitrocellulose membranes. Blots were probed with rabbit polyclonal anti-mouse RelB (Santa Cruz Biotechnology, #sc-226), rabbit polyclonal anti-human Bcl10 (Santa Cruz Biotechnology, #sc-5611), rabbit polyclonal anti-human MALT1 (Santa Cruz Biotechnology, #sc-28246) and mouse monoclonal anti-human CYLD (Santa Cruz Biotechnology, #sc-74435) antibodies. The antibody for detection of cleaved Bcl10 was generated by immunizing rabbits with a peptide N-terminal to the cleavage site (ThermoFisher).

### Flow cytometry

Splenocytes, thymocytes, and peritoneal cavity cells were resuspended in PBS containing 1% FBS and Rat Anti-Mouse CD16/CD32 Mouse BD Fc Block (BD Biosciences). The following antibody combinations were used: anti-CD4-PE (BD Biosciences, clone GK1.5) and anti-CD8a-PerCP (BD Biosciences, clone 53–6.7); anti-IgD-PE (BD Biosciences, clone 11-26c.2a) and anti-IgM-APC (SouthernBiotech, clone 1B4B1); anti-CD5-APC (BD Biosciences, clone 53–7.3) and anti-IgM-PE (SouthernBiotech, clone 1B4B1); and anti-CD21-APC (BD Biosciences, clone 7G6) and anti-CD23-FITC (BD Biosciences, clone B3B4). Flow cytometry was performed with a BD FACSCaliber and Flojo software (Treestar).

### Cellular assays

Splenic lymphocytes were purified using Mouse CD45R (B220) MicroBeads, Mouse CD4^+^ T Cell Isolation Kit II, and Mouse CD8a^+^ T Cell Isolation Kit II (Miltenyi Biotech). For cell activation studies in 96-well plates, 1 x 10^5^ B cells/well were stimulated with 20 μg/ml anti-IgM F(ab’)_2_ (Jackson ImmunoResearch) or 20 μg/ml of ultrapure LPS (InvivoGen), and 5 x 10^4^ cells/well CD4^+^ or CD8^+^ T cells were stimulated with anti-CD3/CD28 Dynabeads (Life Technologies), with or without pretreatment with 126 μM MALT1 inhibitor Z-VRPR-FMK (Enzo Life Sciences). For proliferation assays, cells were stimulated for 72 h at 37°C, pulsed with 1 μCi/well ^3^H-thymidine at 37°C for 24 h, and harvested on a glass fiber plate (Millipore). Cytokine production was measured after 20 h stimulation at 37°C using a electrochemiluminescent Meso Scale Discovery SECTOR Imager 6000 (MSD) and the appropriate single- or multi-plex analyte plates.

For anti-IgM dose response proliferation assays, B220^+^ B cells were labelled with 5-(and 6)-Carboxyfluorescein diacetate succinimidyl ester (CFSE) (C-1157; Invitrogen) and then plated in 96-well round bottom plates. 2 x 10^5^ B cells/well were stimulated with the indicated concentrations of anti-IgM F(ab’)_2_ (Jackson ImmunoResearch). After three days, cells were analyzed for proliferation as determined by CFSE dilution. Cells were also analyzed for survival by propidium iodide staining (BD Bioscience). Flow cytometry was performed as above.

Bone marrow-derived dendritic cells (BMDCs) were prepared by culturing bone marrow cells in 20 ng/ml mouse GM-CSF (Peprotech) for 12 days, and replacing the media every 3 days. BMDCs were purified using CD11c magnetic beads (Miltenyi Biotec), and 1 x 10^5^ cells/well in 96-well plates were stimulated with 100 μg/ml of the Dectin-1 ligand curdlan (InvivoGen) or 10 μg/ml of the Mincle ligand trehalose-6,6-dibehenate (TDB; InvivoGen) for 20 h at 37°C. For Dectin-2 stimulations, 1 x 10^5^ cells/well were incubated with 5 mg/ml anti-mouse Dectin-2 (Miltenyi) for 2 h at 25°C, transferred to plates pre-coated with 10 μg/ml rat anti-Fcγ IgG F(ab’)_2_ (Jackson ImmunoResearch Laboratories), and incubated for 20 h at 37°C. Where appropriate, cells were pretreated with 126 μM Z-VRPR-FMK for 45 min. Culture supernatants were analyzed for cytokine production as above.

### T-dependent and T-independent immunizations

Basal serum Ig levels were determined by ELISA (eBioscience). Immunizations were performed by i.p. injection of 50 μg of keyhole limpet hemocyanin (KLH; EMD) in complete Freund's adjuvant (Sigma) or 25 μg of 2,4,6-trinitrophenyl (TNP)-Ficoll (Biosearch Technologies), and serum samples were collected after 7, 14, 21, and 28 days. KLH- or TNP-specific IgM and IgG levels were determined by ELISA (Life Diagnostics).

### Histopathology

Nineteen different tissues (adipose, adrenal gland, brain, colon, eye, heart, jejunum, kidney, liver, lung, mesenteric lymph node, optic nerve, ovary, pancreas, skeletal muscle, spleen, stomach, testes, and thymus) were collected from 10–15 week old *Wt* (n = 3), *Malt1* ^-/-^ (n = 3), *Malt1* ^PD/PD^ (n = 6), and *Malt1* ^+/PD^ (n = 2) mice, and processed to slides at 5 μm thickness using standard methodology, and stained with hematoxylin and eosin. Histology was evaluated in a blinded fashion by a veterinary pathologist (MAR).

### Immunohistochemistry

Immunohistochemistry (IHC) staining for T cells, B cells and macrophages was performed on formalin-fixed, paraffin-embedded 5 μm tissue sections. Slides were placed on the Ventana Discovery System XT and all reagents, except as noted below, were obtained from Ventana Medical Systems. Briefly, sections were deparaffinized with EZ Prep and subjected to heat antigen retrieval with an EDTA-based buffer CC1 (anti-CD45R and anti-CD3) or Protease I (anti-F4/80). Non-specific staining was blocked with a protein block (Dakocytomation), endogenous biotin (Biotin Blocking Kit) and endogenous peroxidase (Inhibitor D). Primary antibodies against mouse T cells (anti-CD3; DAKO; 1:1000), B cells (anti-CD45R; BDPharmingen; 0.1ug/mL), and macrophages (anti-F4/80; ABDSerotec; 5ug/mL) were applied for 1 hr. An isotype and concentration matched control slide was included as a negative control. Primary antibodies were labeled with either rabbit Ultra MAP-HRP (anti-CD3), rat Ultra-MAP-HRP with 5% normal mouse serum (anti-F4/80) or a mouse-absorbed anti-rat biotinylated secondary antibody (Vector Labs; 1:200) followed by strepavidin-HRP. Immunoreaction was detected with 3’3’-diaminobenzidine (DAB). Slides were counterstained, dehydrated, cleared, and cover-slipped.

In this study, anti-CD45R and anti-CD3 immunostaining were considered to be highly selective in mouse tissues for identifying general B cell or T cell populations, respectively, yielding little to no noticeable background staining. However, IHC markers in mice that permit exclusive macrophage identification from formalin-fixed, paraffin-embedded sections are lacking. This laboratory has found anti-F4/80 to be one of the better commercially available general macrophage markers in mouse tissues. Compared to the antibodies used for lymphocyte identification, our experience has been that anti-F4/80 is much less selective as a macrophage marker and results in a higher background staining of non-histiocytic cells (neutrophils, erythrocytes, and mesenchymal cells). Therefore, reliable interpretation of the anti-F4/80 results for mouse macrophage identification requires additional consideration of cellular staining intensity, cellular/nuclear morphology of stained cells, and taking into account concurrent staining pattern of the mixed inflammatory population with other immunomarkers (anti-CD3 and anti-CD45R).

### Statistics

**Fig 2:**
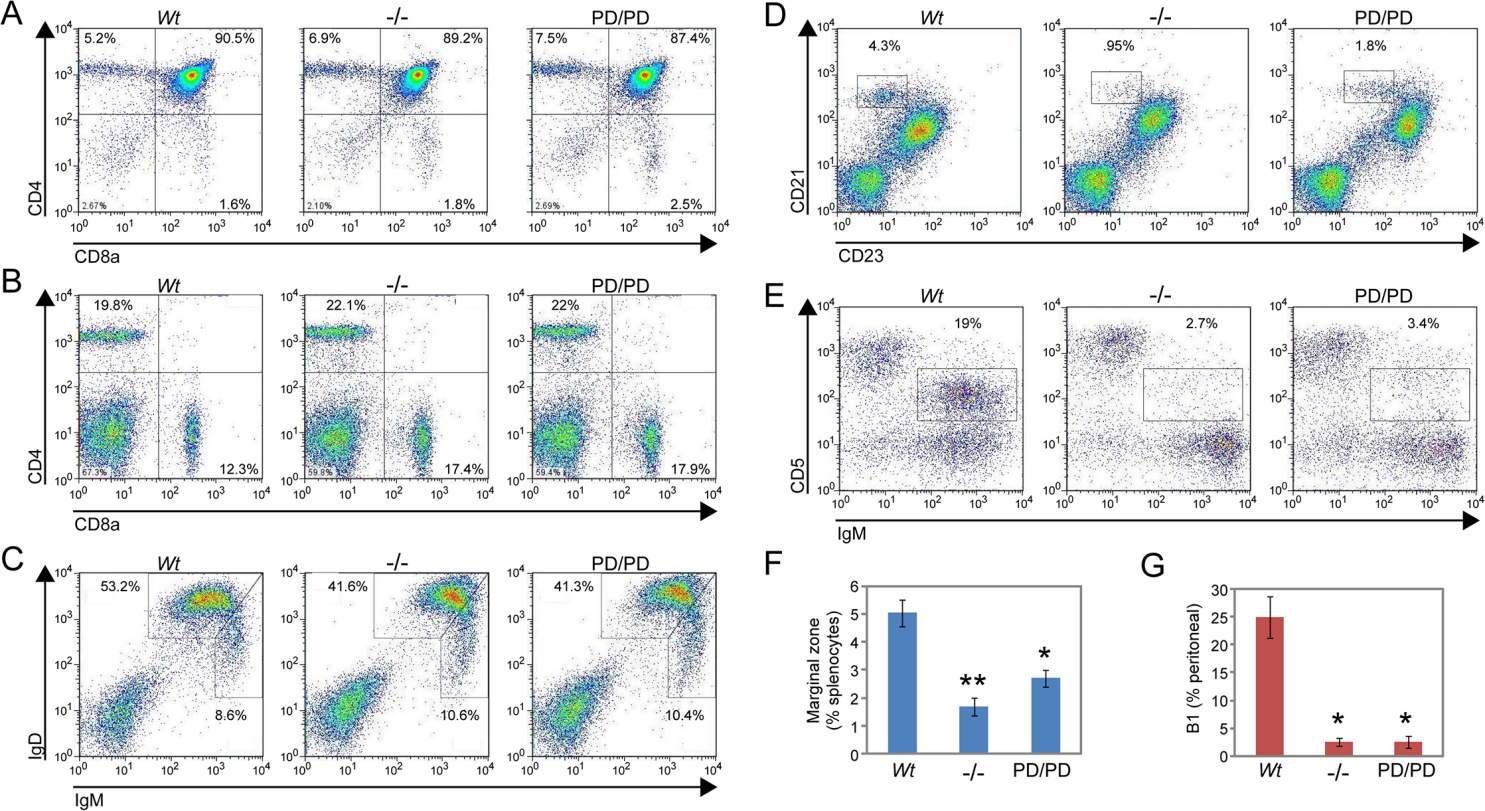
The development of MZ and B1 B cells populations is dependent upon MALT1 protease activity. Representative flow cytometry analysis of lymphocyte populations in MALT1 *Wt*, *Malt1* ^-/-^, and *Malt1* ^PD/PD^ mice. (A) CD4^+^CD8a^+^, CD4^+^CD8a^-^, and CD4^-^CD8a^+^ T cell populations in the thymus. (B) CD4^+^CD8a^+^, CD4^+^CD8a^-^, and CD4^-^CD8a^+^ T cell populations in the spleen. (C) Mature follicular (IgD^hi^IgM^lo^), transitional T1 (IgD^lo^IgM^hi^), and T2 (IgD^hi^IgM^hi^) B cell populations in the spleen. (D, F) MZ B cell (CD21^-^CD23^+^) population in the spleen. (E, G) B1 B cell (IgM^hi^CD5^lo^) population in peritoneal fluid. Bar graphs represent average cell numbers for 3–4 mice/group. Significance was determined relative to the *Wt* groups, with *p < 0.05, **p < 0.01. Error bars represent +/- SEM.

**Fig 3:**
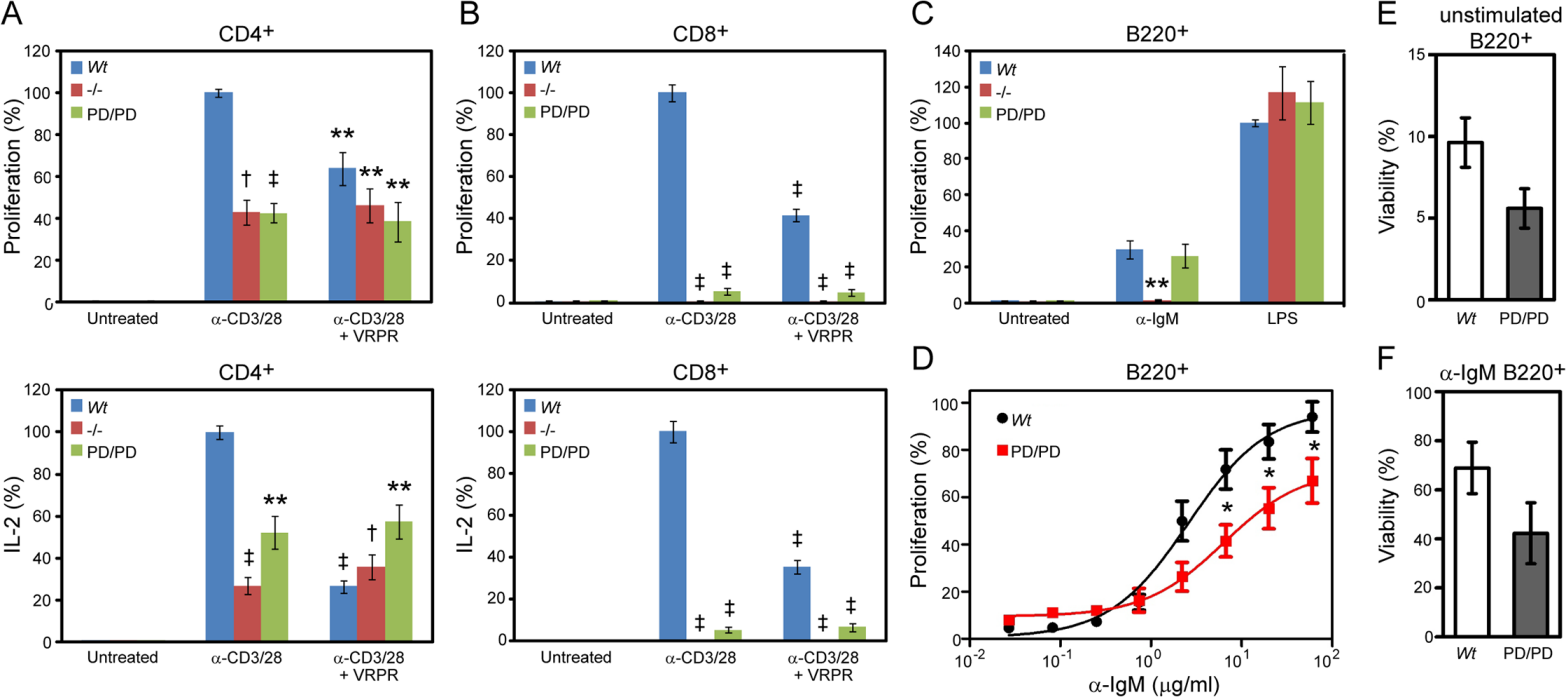
MALT1 protease activity is required for TCR- but not BCR-mediated lymphocyte activation. (A) CD4^+^ T cells and (B) CD8^+^ T cells from the spleens of *Wt*, *Malt1* ^-/-^, and *Malt1* ^PD/PD^ mice were activated by co-stimulation with anti-CD3 and anti-CD28 beads, and proliferation was measured after 96 h by ^3^H-thymidine incorporation, and IL-2 production after 24 h by MSD. The total IL-2 produced by CD4^+^ and CD8^+^ T cells from *Wt* mice was approximately 2–3 ng/ml and 100–400 pg/ml, respectively. Where indicated, T cells were treated with 126 μM of the MALT1 inhibitor Z-VRPR-FMK (VRPR) for 45 min prior to stimulation. (C) B220^+^ B cells from the spleens of *Wt*, *Malt1* ^-/-^, and *Malt1* ^PD/PD^ mice were stimulated with 20 μg/ml anti-IgM or 20 μg/ml LPS and proliferation was measured after 96 h by ^3^H-thymidine incorporation. Bar graphs from Fig 3A-C represent the average response expressed as a percent of the stimulated *Wt* control (LPS treatment for B220^+^ cells) for 4–6 mice/group, and are representative of 3 or more studies. (D) Splenic B220^+^ B cells from *Wt* and *Malt1* ^PD/PD^ mice were labeled with CFSE prior to stimulation with anti-IgM and proliferation was analyzed by FACS three days post stimulation. *Wt* EC50 = 2.6 μg/ml; *Malt1* ^PD/PD^ EC50 = 6.4 μg/ml. Each data point represents the average response of five mice expressed as a percent of *Wt* proliferation at the highest dose. (E) Splenic B220+ B cells were either unstimulated or (F) stimulated with α-IgM (20 μg/ml) for three days and viability was analyzed by FACS. Each bar is an average from five mice. Significance was determined relative to the stimulated *Wt* groups, with *p < 0.05, **p < 0.01, †*p* < 0.001, ‡*p* < 0.0001. Error bars represent +/- SEM.

**Fig 4:**
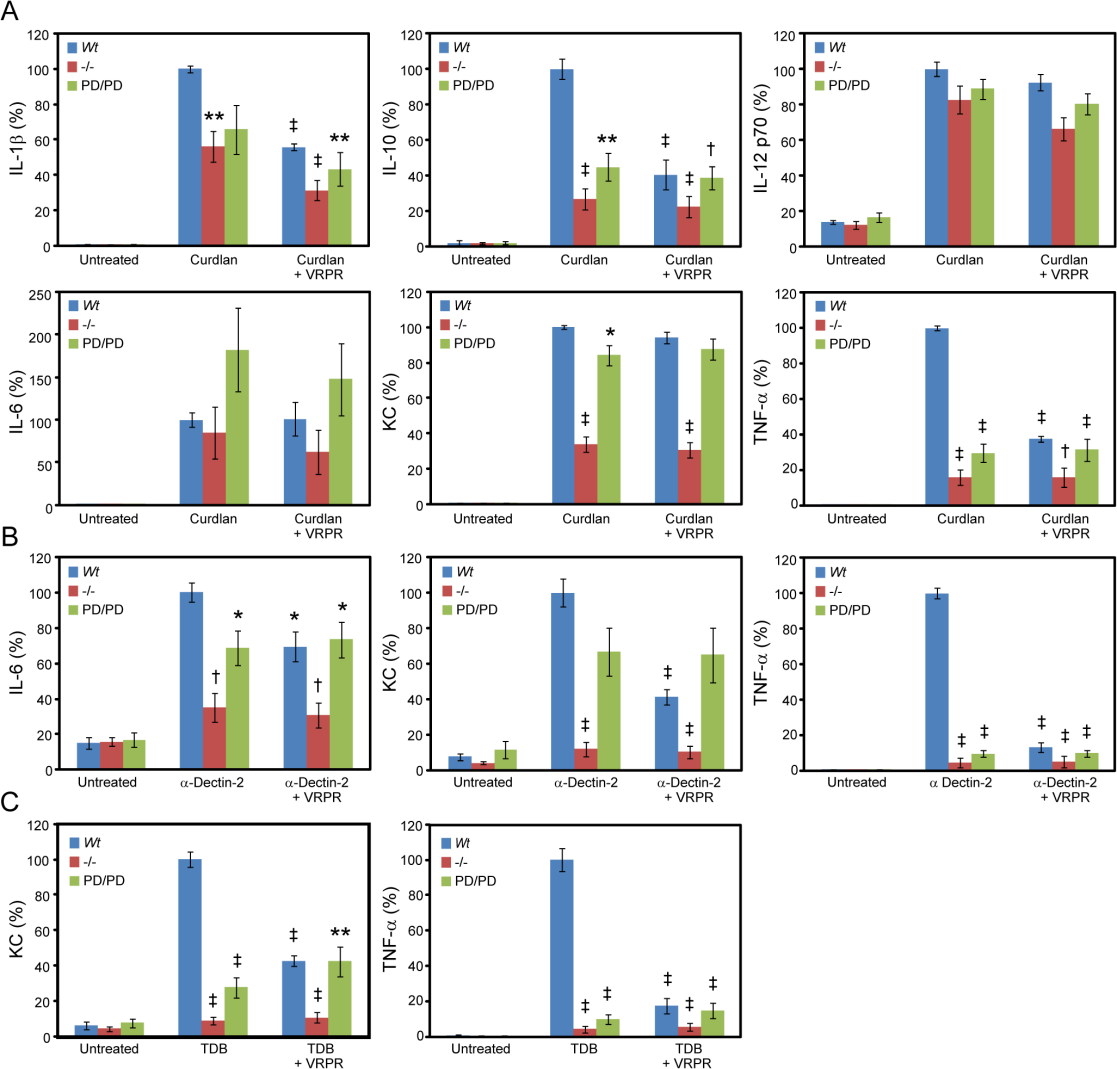
CLR stimulation of cytokine production by BMDCs involves MALT1 protease activity. BMDCs from *Wt*, *Malt1* ^-/-^, and *Malt1* ^PD/PD^ mice were stimulated with (A) curdlan to activate Dectin-1, (B) anti-Dectin-2 antibody to activate Dectin-2, and (C) TDB to activate Mincle. IFN-γ, IL-1β, IL-10, IL-12 p70, IL-6, KC (IL-8 homologue), and TNF-α levels were measured after 20 h by MSD, and results are shown only for cytokines for which significant expression was detected. Maximum cytokine concentration ranges detected were: curdlan, 1–3 ng/ml IL-1β, 4–20 ng/ml IL-6, 4–9 ng/ml KC, 0.5–1 ng/ml IL-10, 0.2–0.4 ng/ml IL-12p70, and 40–100 ng/ml TNF-α; anti-Dectin-2, 0.1–0.3 ng/ml IL-6, 0.5–2 ng/ml KC, and 3–15 ng/ml TNF-α; TDB, 0.3–0.6 ng/ml KC, and 0.3–2 ng/ml TNF-α. Where indicated, cells were treated 126 μM Z-VRPR-FMK (VRPR) for 45 min prior to stimulation. All bar graphs represent the average response expressed as a percent of the stimulated *Wt* control for 6 mice/group, and are representative of 3 or more studies. Significance was determined relative to the stimulated *Wt* groups, with *p < 0.05, **p < 0.01, †*p* < 0.001, ‡*p* < 0.0001. Error bars represent +/- SEM.

**Fig 5:**
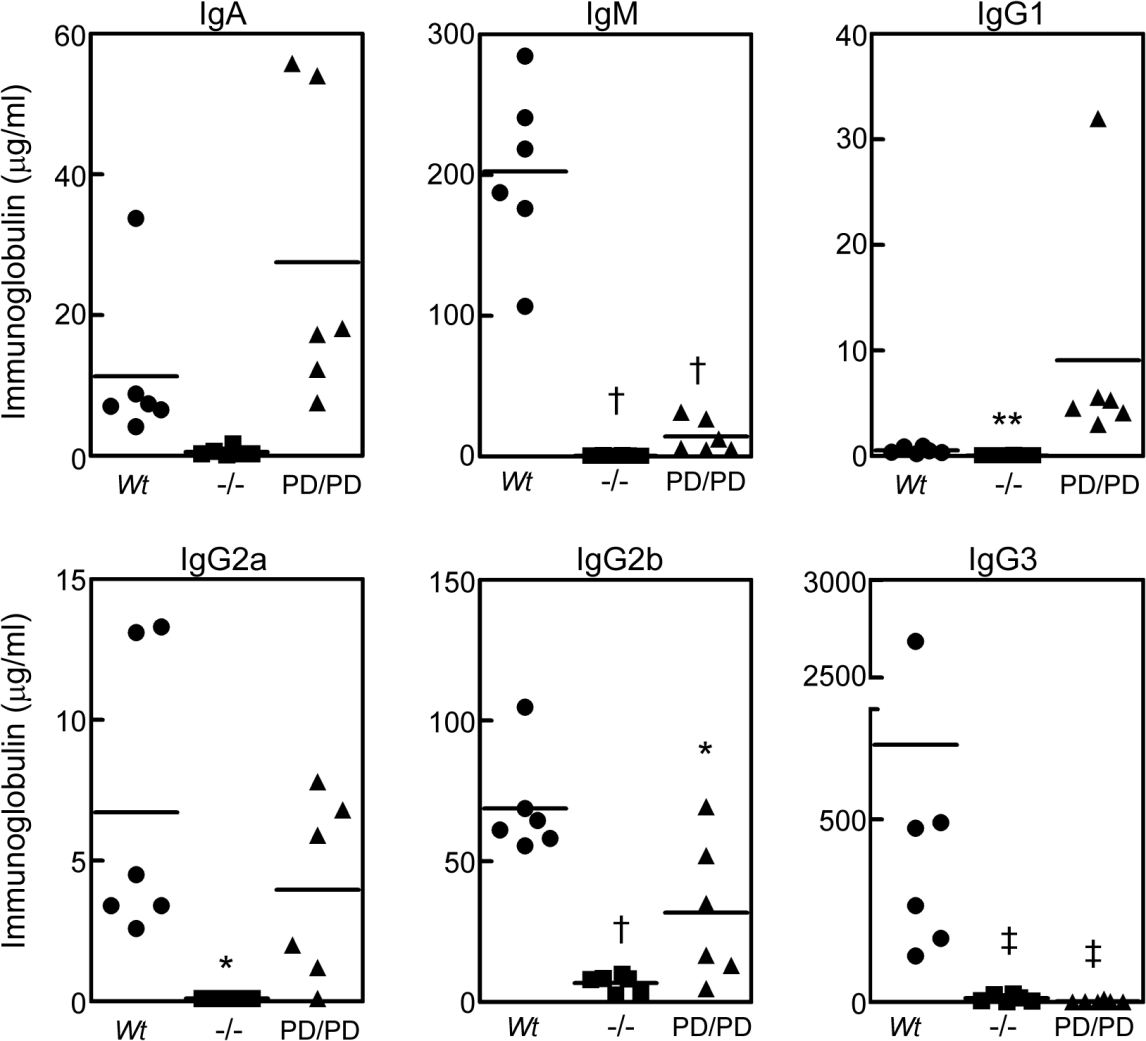
Decreased basal serum Ig levels in MALT1 null and protease-dead mice. ELISA analysis of IgA, IgG1, IgG2a, IgG2b, IgG3, and IgM levels in non-immunized sera from *Wt*, *Malt1* ^-/-^, and *Malt1* ^PD/PD^ mice (n = 6/group). Data represents Ig levels in individual animals, and averages are indicated by horizontal lines. Significance was determined relative to the *Wt* groups, *p < 0.05, **p < 0.01, †*p* < 0.001, ‡*p* < 0.0001.

**Fig 6:**
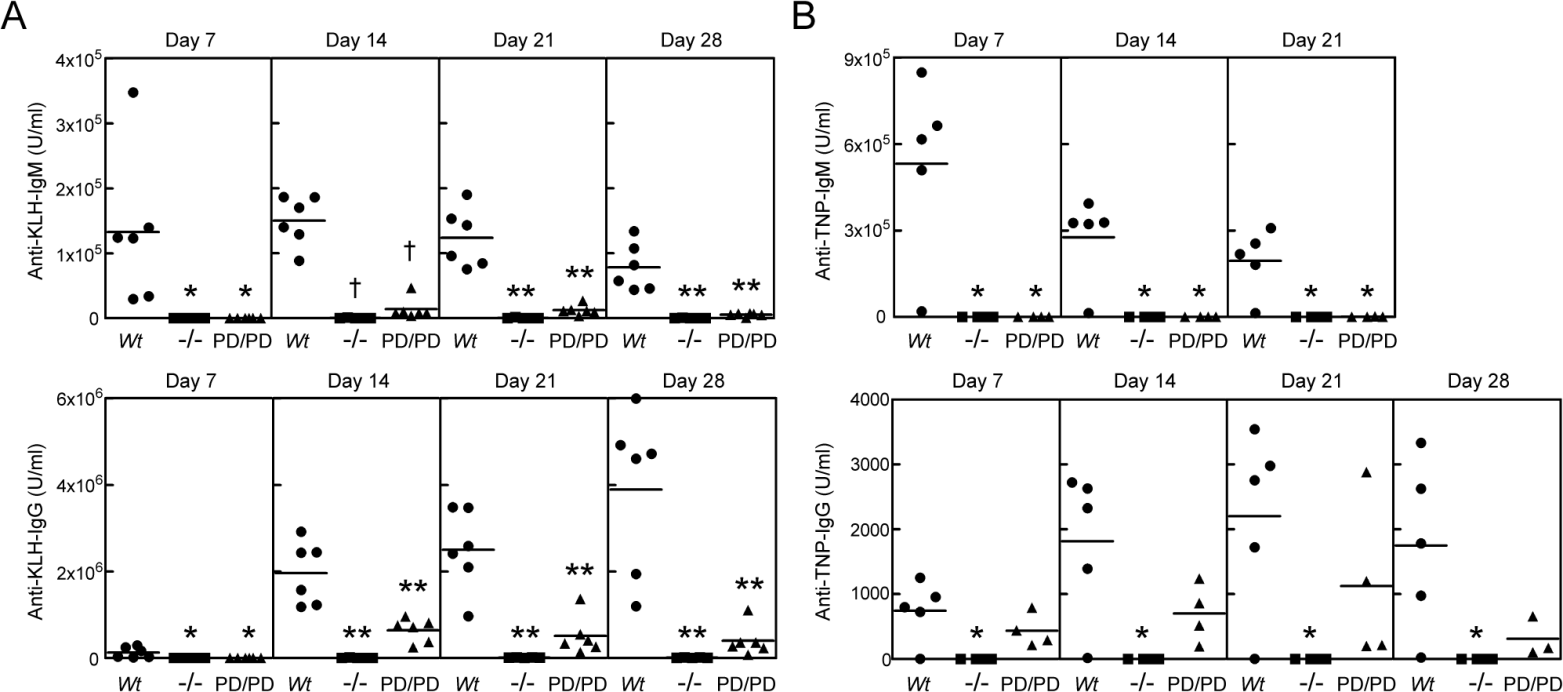
MALT1 protease activity is required for maximal T-dependent and T-independent antibody responses *in vivo*. (A) *Wt*, *Malt1* ^-/-^, and *Malt1* ^PD/PD^ mice (n = 6/group) were immunized with the T-dependent antigen KLH, and serum anti-KLH antibody titers were measured by ELISA on days 7, 14, 21 and 28 after immunization. (B) *Wt*, *Malt1* ^-/-^, and *Malt1* ^PD/PD^ mice (n = 4-5/group) were immunized with the T-independent antigen TNP-Ficoll, and serum anti-TNP antibody titers were measured by ELISA on days 7, 14, 21 and 28 after immunization. Data represents Ig levels in individual animals, and averages are indicated by horizontal lines. Significance was determined relative to the *Wt* groups, with *p < 0.05, **p < 0.01, †*p* < 0.001.

Data were analyzed using JMP 11.0.0 software (SAS Institute). Except for Fig 3D–3F, due to a lack of normality and unequal variances in the data, a log10 transformation was performed on all endpoints. For Fig 2 and Fig 5, an Analysis of Variance (ANOVA) was performed for each log-transformed endpoint using mouse genotype as the factor. For Fig 3A–3C and Fig 4, an ANOVA was performed for each log-transformed endpoint by day using mouse genotype and plate as the factors. For Fig 6, an ANOVA was performed for each log-transformed endpoint by day using mouse genotype as the factor. The *p* values for associated differences of interest were adjusted for multiple comparisons using Tukey’s test. A two-sided t-test was performed to compare *Wt* and Malt1^PD/PD^ for each of the four highest concentrations in Fig 3D and for Fig 3E and 3F. Symbols representing adjusted *p* values are listed in each figure legend. Statistics were performed by a Ph.D.-trained statistician (ADA).

## Results

### Generation of mice with MALT1 protease-dead and null mutations

To examine the role of MALT1 protease activity in immune cells, we generated homozygous *Malt1* ^PD/PD^ protease-dead knockin mice by insertion of a catalytically inactive C472A mutation into exon 12, which encodes the protease active site (Fig 1A). To identify MALT1 functions that are independent of its protease activity, we also generated homozygous *Malt1* ^-/-^ null knockout mice by deletion of exon 12 (Fig 1A). Analysis of total splenic B cells confirmed that MALT1 protein expression (Fig 1B) was similar in *Wt* and *Malt1* ^PD/PD^ mice, but absent in *Malt1* ^-/-^ mice. As shown in Fig 1B and S2 Fig, PMA plus ionomycin stimulation of MALT1 protease activity resulted in the expected cleavage of MALT1 substrates CYLD [31], Bcl10 [25], and RelB [32] in B cells from *Wt* mice, but not *Malt1* ^PD/PD^ mice, confirming that the C472A mutation inactivated MALT1 protease function (S2 Fig—RelB cleavage is impaired in activated B cells expressing MALT1 (C472A)).

### MALT1 protease activity is required for the development of MZ and B1 B cells

To determine if lymphocyte development is dependent upon MALT1 protease function, we examined the abundance of the major T and B cell populations by flow cytometry. As illustrated in Fig 2A and 2B, the CD4^+^CD8a^+^, CD4^+^CD8a^-^, and CD4^-^CD8a^+^ T cell populations in the thymus and spleen were unchanged between *Wt*, *Malt1* ^-/-^, and *Malt1* ^PD/PD^ mice. Similarly, mature follicular B cell (IgD^hi^IgM^lo^), transitional T1 B cell (IgD^lo^IgM^hi^), and transitional T2 B cell (IgD^hi^IgM^hi^) populations in the spleen were indistinguishable across all three genotypes (Fig 2C). However, despite normal follicular B cell development, both *Malt1* ^-/-^ and *Malt1* ^PD/PD^ mice exhibited significantly decreased MZ B cells (CD21^-^CD23^+^) in the spleen (Fig 2D and 2F). The B1 B cell (IgM^hi^CD5^lo^) population was also almost completely absent in peritoneal fluid of *Malt1* ^-/-^ and *Malt1* ^PD/PD^ mice (Fig 2E and 2G). These results are consistent with those previously reported for *Malt1* ^-/-^ mice [14,15], and indicate that MALT1 is not required for T cell and follicular B cell maturation in the spleen. However, MALT1 protease activity is crucial for the development of the innate-like MZ and B1 B cell populations, which are known to rapidly respond to blood-borne antigens in a T cell-independent manner [33].

### Antigen receptor-mediated activation of T cells, but not B cells, is dependent on MALT1 protease activity

We next examined the role of MALT1 protease activity in the *ex vivo* activation of T and B cells. CD4^+^ and CD8^+^ T cells were purified from the spleens of *Wt*, *Malt1* ^-/-^, and *Malt1* ^PD/PD^ mice, and co-stimulated with bead-bound anti-CD3 and anti-CD28. As shown in Fig 3A and 3B, TCR activation induced robust proliferation and IL-2 production by CD4^+^ and CD8^+^ T cells from *Wt* mice. In contrast, CD4^+^ T cells from *Malt1* ^-/-^ or *Malt1* ^PD/PD^ mice displayed ~2-fold reduced proliferation and IL-2 production (Fig 3A), whereas little or no response was detected for CD8^+^ T cells from these mice (Fig 3B). These results are consistent with those previously reported for total T cells from *Malt1* ^-/-^ mice [14,15], although others have observed only a minimal effect of MALT1 deficiency on anti-TCRβ-stimulated proliferation of CD8^+^ T cells [34], which is a stronger stimulus than provided by the anti-CD3/CD28 stimulation used herein. Decreased proliferation was also observed following pretreatment of CD4^+^ and CD8^+^ T cells from *Wt* mice with the MALT1 inhibitor Z-VRPR-FMK (Fig 3A and 3B), although in many experiments we found that the MALT1 inhibitor treatment tended to result in a weaker effect than was observed in *Malt1* ^PD/PD^ mice, consistent with its known poor potency in cellular assays. These findings demonstrate that MALT1 protease activity plays an important role in TCR-mediated T cell activation.

B220^+^ B cells were also purified from the spleens of *Wt*, *Malt1* ^-/-^, and *Malt1* ^PD/PD^ mice, and stimulated *ex vivo* with a high dose of anti-IgM. As shown in Fig 3C, B cells from *Malt1* ^PD/PD^ and *Wt* mice displayed similar levels of proliferation (Fig 3C). In contrast, B cells from *Malt1* ^-/-^ mice had severely reduced proliferation in response to B cell receptor (BCR) ligation, demonstrating that MALT1 is required for BCR-mediated B cell proliferation, although this has been controversial [14,15], while its protease activity appears not. One interpretation of this result is that BCR induces proliferation independently of MALT1 protease activity at high agonist concentrations. However, it is possible that MALT1 protease activity contributes to responses at lower agonist doses. To address this possibility, we performed anti-IgM dose reponse studies with *Malt1* ^PD/PD^ and *Wt* B cells. We found that *Malt1* ^PD/PD^ B cells are partially defective in achieving a maximal proliferative response and require two to three-fold increase in effective dose to achieve 50% maximal proliferation (EC50) (Fig 3D). In addition to evaluating proliferation, we also examined MALT1 protease contribution to B cell survival in unstimulated and BCR stimulated cells. Although *Malt1* ^PD/PD^ B cells appeared slightly less viable than *Wt* cells after three days of culture in each condition, we observed no statistical difference in survival rates between both genotypes (Fig 3E and 3F). Overall, these findings support our view that MALT1 protease dependent and independent activites contribute to optimal proliferative signaling.

Since conflicting results have been reported regarding the role of MALT1 in the response of B cells to stimulation with bacterial LPS [14,15], we also examined LPS-stimulated proliferation of B220^+^ B cells from *Wt*, *Malt1* ^-/-^, and *Malt1* ^PD/PD^ mice. However, as shown in Fig 3C, no differences were observed in the LPS response of B cells across all three genotypes. Taken together, these results demonstrate that MALT1 protease activity plays an important role in signaling through the TCR and a more subtle role in signaling following BCR stimulation.

### CLR-stimulated cytokine production by dendritic cells involves MALT1 protease activity

CBM complexes have been implicated in receptor-mediated signal transduction in a variety of non-lymphocyte cell types, including through CLRs in dendritic cells [6,7,18,19]. To evaluate the role of MALT1 protease activity in CLR signaling, we differentiated BMDCs from bone marrow precursors isolated from *Wt*, *Malt1* ^-/-^, and *Malt1* ^PD/PD^ mice, stimulated them with different CLR ligands, and assessed the production of a panel of cytokines. Simulation of BMDCs from *Wt* mice with the Dectin-1 ligand curdlan induced production of IL-1β, IL-10, IL-12 p70, IL-6, KC (IL-8 homologue), and TNF-α (Fig 4A). Of these cytokines, IL-10 and TNF-α were significantly reduced in curdlan-stimulated BMDCs from both *Malt1* ^-/-^ and *Malt1* ^PD/PD^ mice, and there was a trend towards reduced IL-1β (*p* = 0.06) (Fig 4A). In contrast, KC production was dramatically reduced only in BMDCs from *Malt1* ^-/-^ but not *Malt1* ^PD/PD^ mice, whereas IL-6 and IL-12 p70 were not decreased irrespective of genotype (Fig 4A). Consistent with these results, pre-treatment of BMDCs from *Wt* mice with Z-VRPR-FMK also inhibited production of IL-1β, IL-10, and TNF-α, but not IL-6, KC, or IL-12 p70 (Fig 4A).

We also examined BMDC cytokine production stimulated through the Dectin-2 and Mincle CLRs using an anti-Dectin-2 antibody and TBD as ligands, respectively. In BMDCs from *Wt* mice, anti-Dectin-2 stimulated production of only IL-6, KC, and TNF-α, while TBD stimulated production of only KC and TNF-α (Fig 4B and 4C). For Dectin-2 signaling, IL-6 and TNF-α production was decreased in BMDCs from both *Malt1* ^-/-^ and *Malt1* ^PD/PD^ mice, while KC was significantly decreased only in *Malt1* ^-/-^ cells. Similarly, for Mincle signaling, KC and TNF-α production was also decreased in BMDCs from both *Malt1* ^-/-^ and *Malt1* ^PD/PD^ mice (Fig 4C). Treatment with Z-VRPR-FMK gave results similar to those described above for Dectin-2 and Mincle (Fig 4B and 4C). These results indicate that the production of some, but not all cytokines following stimulation of CLRs in dendritic cells is dependent upon MALT1 protease activity.

### MALT1 protease activity is required for T-dependent and T-independent immune responses in vivo

The above results indicate that some, but not all cellular functions that are mediated by CBM complexes are dependent upon MALT1 protease activity in *ex vivo* cultured immune cells, whereas others are likely only dependent on MALT1 scaffolding activity, or are entirely MALT1-independent. To explore how these aspects of MALT1 function are integrated in the more complex *in vivo* setting, we next examined the role of MALT1 protease activity in immune responses in mice. Consistent with previous results [14,15], naive *Malt1* ^-/-^ mice displayed severely reduced basal serum Ig levels (Fig 5). *Malt1* ^PD/PD^ mice, while having significantly reduced serum IgM, IgG2b, and IgG3 levels, were relatively normal with respect to other Ig isotypes (Fig 5).

We next examined the *in vivo* antibody responses of *Wt*, *Malt1* ^-/-^, and *Malt1* ^PD/PD^ mice to immunization with T cell-dependent and-independent antigens. When immunized with the T-dependent antigen KLH, *Wt* mice produced high titers of anti-KLH IgM from days 7 through 28, and the expected Ig class switch occurred by day 14, with the anti-KLH IgG concentration increasing through day 28 (Fig 6A). As previously reported [14,15], *Malt1* ^-/-^ mice were unable to mount anti-KLH IgM and IgG responses (Fig 6A). Similarly, *Malt1* ^PD/PD^ mice also displayed poor IgM and IgG responses to KLH immunization, although this effect was not as dramatic as observed in *Malt1* ^-/-^ mice (Fig 6A). Immunization of *Wt*, *Malt1* ^-/-^, and *Malt1* ^PD/PD^ mice with the T-independent type II antigen TNP-Ficoll yielded a similar IgM profile to what was observed for T-dependent immunizations, however, the IgG response of *Malt1* ^PD/PD^ mice was not significantly affected (Fig 6B). These results demonstrate that MALT1 protease activity contributes to T-dependent and T-independent antibody responses *in vivo*.

### MALT1 protease-dead mice develop inflammatory cell infiltrates in multiple organs

**Fig 7:**
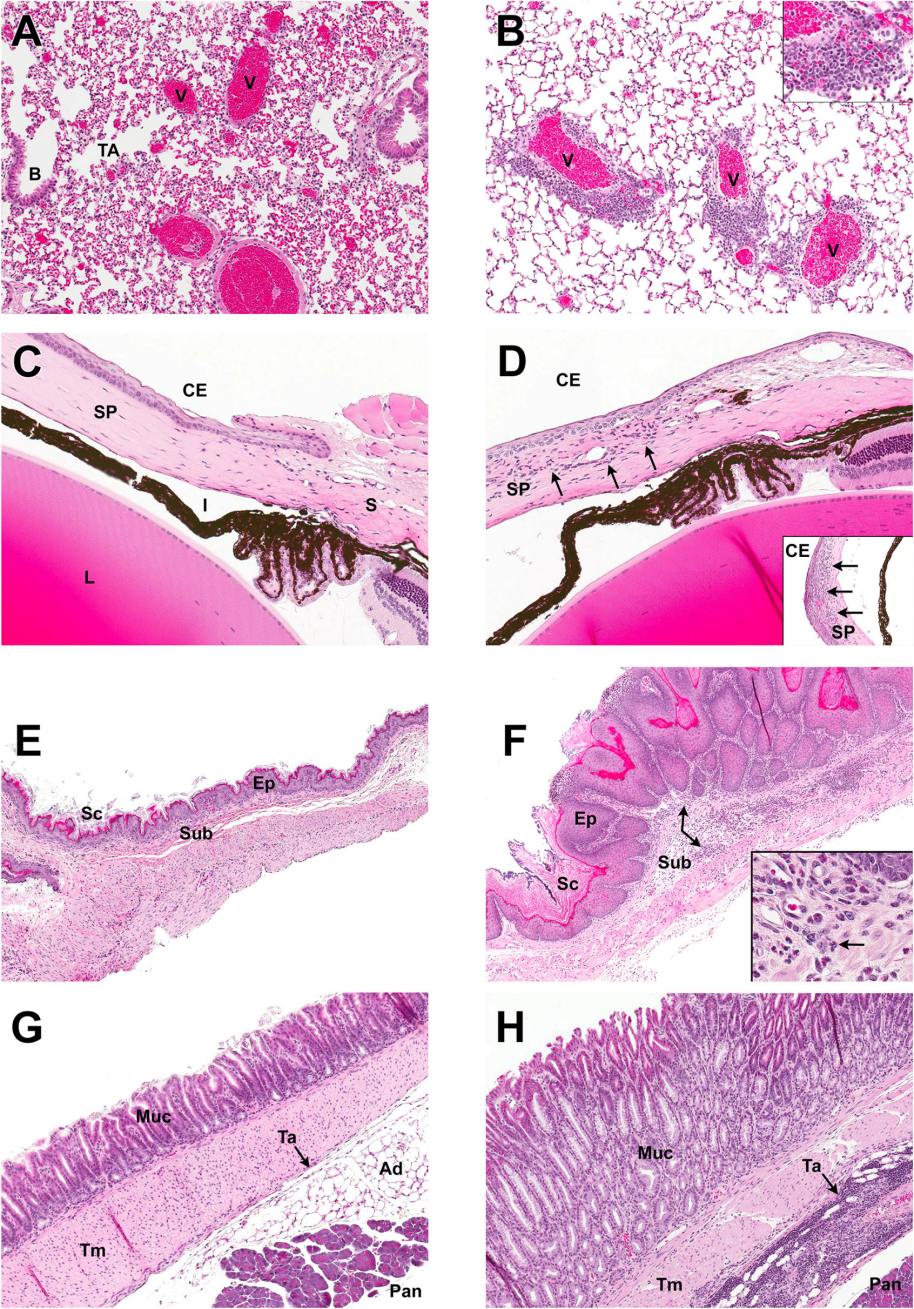
MALT1 protease-dead mice develop inflammatory cell infiltrates in multiple organs. H&E-stained histological sections of tissues from MALT1 *Wt* (left panels A, C, E, G; n = 3) and *Malt1* ^PD/PD^ (right panels B, D, F H; n = 6) mice. Results from *Malt1* ^-/-^ (n = 3) and *Malt1* ^+/PD^ (n = 2) mice were identical to *Wt* mice. (A, B) Lung at 100X magnification. Airways of *Wt* and *Malt1* ^PD/PD^ mice, including the B, bronchiolar (B) and terminal airways (TA) were relatively clear of inflammatory infiltrates. Distinctive findings were present in the lungs of *Malt1* ^PD/PD^ mice, which featured pulmonary veins (V) surrounded by a prominent mixed mononuclear inflammatory cell infiltrate predominated by lymphocytes and histiocytic cells (inset, 400X magnification). (C, D) Limbic region of eye (150X magnification). Cornea from *Wt* eye demonstrates typical anatomical features of a thin corneal epithelium (CE) overlying the substantia propria (SP). Occasional linear nuclei in the SP are part of the normal fibroblast populations. Sclera (S), pigmented iris (I) and lens (L) are normal in *Malt1* ^PD/PD^ mice. Cornea from *Malt1* ^PD/PD^ mice consistently had neutrophilic inflammatory cell infiltrates (arrows) of varying severity in the substantia propria of the cornea. This particular *Malt1* ^PD/PD^ mouse also had a florid neutrophilic infiltrate towards the central portion of the cornea (inset, 150X magnification). (E, F) Non-glandular stomach (50X magnification). Normal non-glandular stomach was comprised of stratified squamous epithelium (Ep) with a small amount of keratinized stratum corneum (Sc) and only occasional inflammatory cells evident in the submucosa (Sub). Non-glandular stomach from *Malt1* ^PD/PD^ mice demonstrated marked hyperkeratosis of the stratum corneum and prominent hyperplasia of the epithelium. A mixed inflammatory infiltrate was commonly present in the mural wall, with this particular mouse having a moderate neutrophilic infiltrate in the submucosa (arrow). (G, H) Glandular stomach (50X magnification). The fundic mucosa (Muc) of the *Wt* mouse is regular, even, and is formed by undulating gastric pits. The relatively acellular tunica adventitia (Ta) is the thin tissue layer (arrow) sandwiched between the thicker tunica muscularis (Tm) and the adipose tissue (Ad) / pancreas (Pan). Reactive hyperplasia of the glandular mucosa was present in *Malt1* ^PD/PD^ mice, and characterized by prominent thickening of the mucosa and loss of regular and even mucosal architecture. Mixed inflammatory cell infiltrates were increased in the mural wall of stomachs from *Malt1* ^PD/PD^ mice, with this particular mouse demonstrating an intense inflammatory infiltrate of the tunica adventicia that is predominated by populations of lymphocytes and histiocytic cells.

*Malt1* ^PD/PD^ mice did not display any externally obvious differences from *Wt* and *Malt1* ^-/-^ mice at weaning. However, as *Malt1* ^PD/PD^ mice aged, we noted that they gradually developed a hunched appearance, behaved in a lethargic manner, and eventually developed clinically progressive hind limb paresis, necessitating humane euthanasia. By 22 weeks of age, 100% morbidity was observed in a cohort of 20 male and female *Malt1* ^PD/PD^ mice, whereas none of these phenotypes were observed in *Wt* or *Malt1* ^-/-^ mice of similar age. To determine the basis for this morbidity, we performed a histological screen of 19 different tissues from *Wt*, *Malt1* ^-/-^, *Malt1* ^PD/PD^, and *Malt1* ^+/PD^ mice. We observed distinct histopathological phenotypes only in *Malt1* ^PD/PD^ mice, most commonly in the lung, eye, and stomach (Fig 7A–7H), and more infrequently in a variety of other tissues, such as brown and white adipose, liver, skeletal muscle, and testes. The lungs of *Malt1* ^PD/PD^ mice consistently displayed vasculocentric mononuclear inflammatory cell infiltrates that were comprised predominately of lymphocytes with fewer histiocytic cells (Fig 7A and 7B). Immune infiltrates were confined mostly to larger pulmonary veins, with occasional penetration into the cardiac muscle layer. The eyes in a majority of the *Malt1* ^PD/PD^ mice also contained a neutrophilic infiltrate within the substantia propria in the limbic region of the cornea (Fig 7C and 7D). The non-glandular portion of the stomach of all *Malt1* ^PD/PD^ mice displayed minimal to marked hyperkeratosis of the stratum corneum and/or hyperplasia of the stratified squamous epithelium (Fig 7E and 7F). Mixed inflammatory infiltrates were also observed in the mural wall of the glandular region of stomach in *Malt1* ^PD/PD^ mice (Fig 7G and 7H).

**Fig 8:**
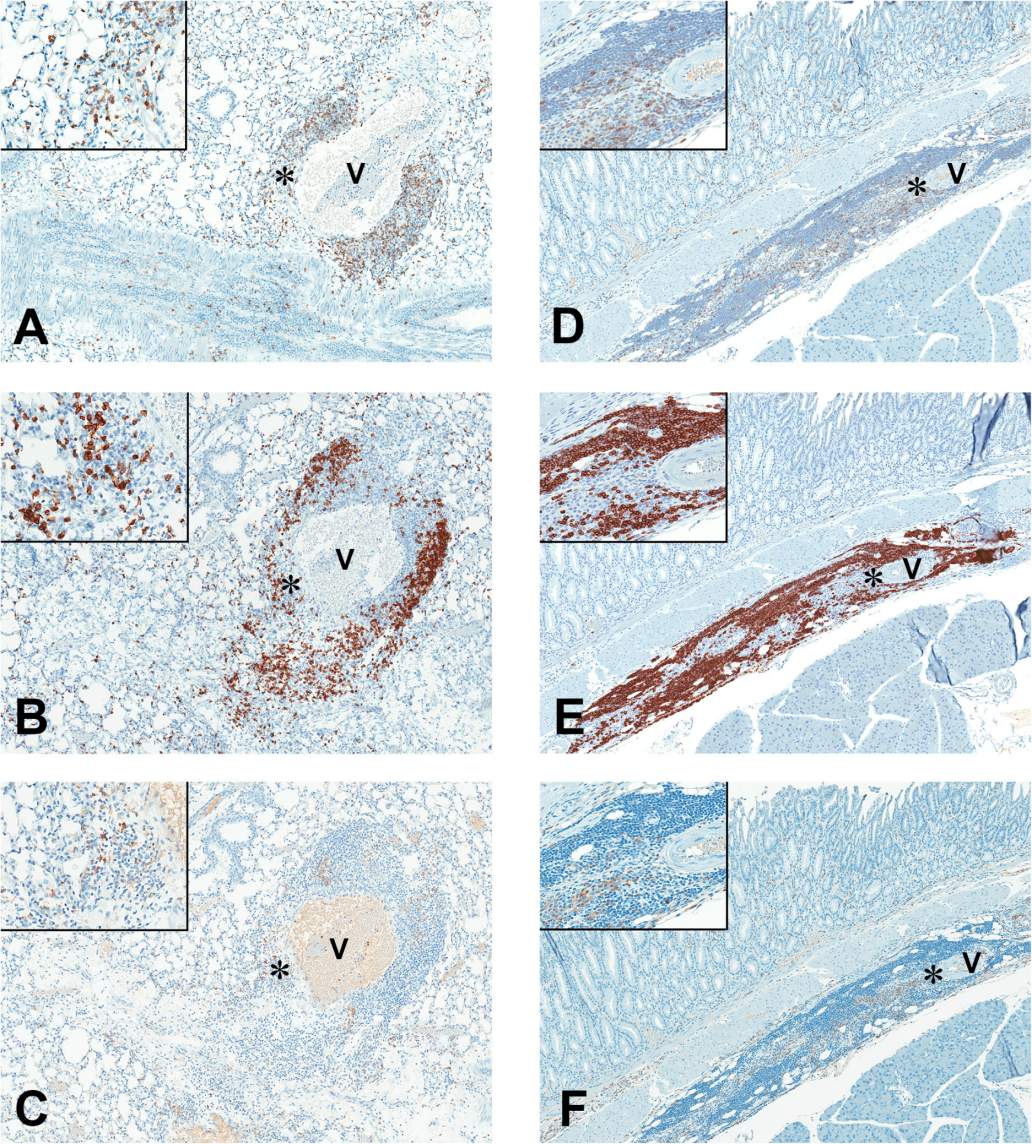
Mixed inflammatory infiltrates of T cells, B cells, and macrophages in MALT1 protease-dead mice. IHC photomicrographs (80X magnification, insets 400X magnification) of inflammatory cell infiltrates surrounding vessels (V) of the lung and tunica adventicia of glandular stomach in *Malt1* ^PD/PD^ mice, using antibodies against: (A, D) CD3 to detect T cells; (B, E) CD45 to detect B-cell; and (C, F) F4/80 to detect macrophage. Non-selective background staining was higher with the F4/80 antibody, as evident with the pale staining of erythrocytes within the vessel lumen (V, Fig 8C). Therefore, macrophage identification was made by considering the higher staining intensity and morphology of cells stained (inset, Fig 8C). IHC photomicrographs of inflammatory cell infiltrates in lung of *Malt1* ^PD/PD^ mice (8A, C) demonstrates a mixed population of T-cells, B-cells and macrophages comprising the perivascular cuffs of pulmonary vessels. Similar to the lung infiltrates of *Malt1* ^PD/PD^, inflammatory infiltrates in deeper glandular portions of stomach had mixed population of T cells, B cells and macrophages in the tunica adventicia (Fig 8D, F).

Additional lung and stomach sections from *Malt1* ^PD/PD^ mice with notable findings were evaluated by IHC to characterize the mononuclear cell populations of the inflammatory infiltrates. In this study, the noted lung infiltrates of *Malt1* ^PD/PD^ mice that were often surrounding pulmonary vessels, were considered to be comprised of a mixed mononuclear population of T cells, B cells, and macrophages based on IHC staining (Fig 8A–8C). In stomachs, there was a tendency for the inflammatory infiltrates in *Malt1* ^PD/PD^ mice to vary with anatomical region affected. Inflammatory infiltrates subadjacent to regions of hyperplastic epithelium in non-glandular stomach were mixed, but dominated by neutrophilic populations, readily identified on H&E sections by their multilobate, polymorphonuclear feature (inset, Fig 7F). In affected mice with deeper, mural infiltrates (tunica adventitia) in glandular portions of stomach, the inflammatory infiltrates were of a mixed mononuclear nature. IHC of these regions in these affected areas demonstrated mixed populations of T cells, B cells, and macrophages, similar to the perivascular infiltrates in lung of the *Malt1* ^PD/PD^ mice (Fig 8D–8F).

Although the exact cause of the clinical demise in *Malt1* ^PD/PD^ mice is unclear, it is likely that the presence of multi-organ inflammatory infiltrates was a contributing factor.

## Discussion

It has become increasingly clear that a wide variety of different receptor families signal through CBM complexes to activate NF-κB [1]. In these different contexts, MALT1 is thought to contribute to NF-κB activation through a combination of both its scaffolding and protease activities. Although studies examining TCR signaling have established a link between MALT1 scaffolding and IKK activation, the relative contribution of MALT1 protease activity (which does not impact IKK activation) has not been well characterized or examined in an *in vivo* context due to the poor pharmacologic properties of available MALT1 inhibitors. By comparing mice homozygous for either null or catalytically inactive mutations in MALT1, we found that many, but not all MALT1 functions were dependent upon its protease activity, and that MALT1 protease activity plays a crucial role that complements its scaffolding activity across multiple immune cell types and in immune responses *in vivo*. In the absence of MALT1 protease activity, CD4^+^ and CD8^+^ T cells displayed decreased activation-induced proliferation and IL-2 production while B cell receptor stimulated proliferation was partially dependent on protease activity. In dendritic cells, stimulation of cytokine production through the Dectin-1, Dectin-2, and Mincle CLRs was also found to be partially dependent upon protease activity. Protease-dead mice displayed reduced basal immunoglobulin levels, and defective T-dependent and T-independent antibody responses. Despite these generally decreased responses, *Malt1* ^PD/PD^ mice, but not *Malt1* ^-/-^ mice, progressively developed inflammatory cell infiltrates in multiple organs, ultimately leading to morbidity due to hind limb paresis. These findings demonstrate the importance of MALT1 protease activity in mounting effective immune responses and in maintaining immune homeostasis *in vivo*.

In lymphocytes, we found that while MALT1 protease function alone was required for optimal TCR activation of CD4^+^ and CD8^+^ T cells, protease dependent and independent activities were required for BCR ligation-induced B cell activation. In the context of isolated cellular responses, these data lead to a number of interesting interpretations. First, the scaffolding activity of MALT1 is not necessarily intertwined with its protease activity, and it is sufficient to dictate a specific set of cellular responses. Likewise, it is formally possible that MALT1 protease activity may also function in isolation to direct a completely different set of responses, although this cannot be determined by comparing *Malt1* ^-/-^ and *Malt1* ^PD/PD^ mice. We also cannot exclude the possibility that in alternate cell types MALT1 protease activity may act strictly in tandem with scaffolding activity to achieve a combined threshold capable of stimulating downstream responses. However, it is apparent that the relative importance of MALT1 scaffolding and protease activities can vary substantially between different receptors and cell types.

In general, these interpretations also extend to the role of MALT1 in innate immune responses to fungal-derived carbohydrates that signal through CLRs. We find that in isolated dendritic cells, depending upon the specific CLRs and cytokines that were examined, cytokine responses can be: MALT1 protease-activity dependent; MALT1-dependent, but protease activity-independent; or entirely MALT1-independent. For example, activation of Dectin-1 induces a range of cytokines, including: KC, IL-10, and TNF-α. While we found IL-10 and TNF-α to be MALT1 protease activity-dependent cytokines, KC appeared to be dependent only on MALT1 scaffolding activity. Similar differential requirements for either MALT1 scaffolding or protease activities were also seen for Dectin-2 and Mincle. The composite effect of the outputs mediated by MALT1 scaffolding and protease activities in lymphocytes and dendritic cells is likely to culminate in optimally orchestrated immune responses *in vivo*.

The importance of MALT1 protease activity in an *in vivo* context is highlighted by our observation that *Malt1* ^PD/PD^ mice display diminished basal serum IgM, IgG2b, and IgG3 levels, although the decrease in serum Ig was more pronounced in *Malt1* ^-/-^ mice. This finding is mirrored by the fact that MALT1 protease activity is also required for the maintenance of innate-like MZ and B1 B cell populations, of which B1 B cells are notable for producing the majority of serum IgM and IgG3 [35]. While untested in our study, MALT1 protease activity may maintain these populations by contributing to survival signals or activation induced proliferation. By contrast, MALT1 scaffolding activity serves a reciprocal role by maintaining appropriate basal levels of all other Ig isotypes. Although it is not clear how these basal serum Ig levels are maintained, tonic (unstimulated) or stimulated BCR signaling via MALT1 scaffolding activity may be a contributing factor.

As yet, we do not have a well-defined mechanistic view of all MALT1 activities across different cellular contexts. Indeed, a complicating factor stems from the different modes by which MALT1 has been shown to activate NF-κB. For example, in TCR signaling, MALT1 is responsible for activating all relevant NF-κB family members, including RelA and c-Rel [14,15]. In contrast, others have reported that during BCR signaling MALT1 only activates c-Rel, while Bcl10 activates RelA in a MALT1-independent manner [17]. As a consequence, MALT1 is not required for RelA-mediated B cell proliferation, but is required for c-Rel survival mechanisms [15,17]. For reasons that are currently unclear, our observation that MALT1 is required for BCR-induced proliferation is not consistent with these findings. Despite this discrepancy, the view that MALT1 selectively activates c-Rel in specific cell types is gaining support. Indeed, a recent examination of Dectin-1 and Dectin-2 signaling in human dendritic cells has also linked MALT1 exclusively to c-Rel, and further identified a critical role for MALT1 protease activity [7]. Given these similar observations involving disparate receptor classes and cell types, it is highly probable that MALT1 protease-mediated c-Rel activation will emerge as a common theme.

Unexpectedly, despite the generally decreased cellular and *in vivo* responses we observed in *Malt1* ^PD/PD^ mice, these animals also developed inflammatory cell infiltrates in multiple organs. Although this phenotype has the potential to confound the analysis of immune cell populations in *Malt1* ^PD/PD^ mice, it is important to note the concordance between our results with protease-dead mice and treatment of *Wt* cells with a MALT1 protease inhibitor. The epithelial pathology (non-glandular stomach) and inflammatory histomorphologic phenotype (lung and stomach) associated with the *Malt1* ^PD/PD^ mice is intriguing, given the minor histomorphological phenotypes previously described with *Malt1* ^-/-^ knockout. Based on the similar makeup of mononuclear inflammatory infiltrate surrounding vessels of both the lung and stomach, it is tempting to posit a causal link between the histopathology and the protease dysfunction in these mice. Considering the function of the gene in B cell orchestration, coupled with the nature and location of the infiltrates, two immediate mechanisms of pathogenesis are worthy of considering, with regards to *Malt1* ^PD/PD^ alteration: aberrant/ectopic mucosa-associated lymphoid tissue formation; and immune mediated (dys)function. A more thorough characterization of *Malt1* ^PD/PD^ pathology will be required, along with functional *in vivo* assessments of gene perturbations, to firmly establish any causal link between the MALT1 protease function and the pathology observed in *Malt1* ^PD/PD^ mice. The underlying reason why this pathology is entirely absent from *Malt1* ^-/-^ mice is not clear and will also require further study. However, this does suggest that MALT1 scaffolding and protease activities must be in balance to support normal immune homeostasis *in vivo*. Given the growing interest in developing selective MALT1 inhibitors for treatment of autoimmune disorders and other diseases [2,3,36–38], it will be important to further explore this potential negative consequence of inhibiting MALT1 protease activity.

This work, along with two papers published during our review process [39,40], represent a key step in uncovering the physiological contributions of MALT1 scaffolding and protease activities in innate and adaptive immunity. Collectively, we and others identify MALT1 protease activity as a critical determinant for development of MZ and B1 B cells and control of autoimmune inflammation [39,40]. Surprisingly the role of MALT1 protease activity in lymphocyte activation is not consistent across all studies; however, work from our group and Thome’s group [39] strongly support the notion that MALT1 protease activity is critical for adaptive and innate immune cell responses. As outlined above, these results reinforce our view that MALT1 protease activity does not merely adjust IKK mediated NF-κB activation, but contributes significantly at the cellular and organism level to maintain normal systemic immunity. A key remaining question revolves around the mechanism by which MALT1 scaffolding and protease activities integrate signaling downstream of different receptors in different cell types. The identification of additional MALT1 proteolytic substrates will undoubtedly reveal alternate pathways that are regulated by MALT1, such as the cleavage of CYLD and its regulation of JNK [31]. MALT1 protease-dead mice should also prove useful to explore the role of MALT1 in other cellular contexts.

## Supporting Information

S1 Fig Complete western blots confirming MALT1 (C472A) expression and loss of protease activity. Purified total B cells from the spleens of *Wt*, *Malt1* ^-/-^, and *Malt1* ^PD/PD^ mice were treated with (+) or without (-) PMA plus ionomycin for 1 h and assessed by Western blotting for expression of MALT1, CYLD, Bcl10, and proteolytically cleaved CYLD and Bcl10. All samples were run on the same western blot. A single blot was probed sequentially for MALT1 and CYLD. A duplicate blot with the same samples was probed for Bcl10 and cleaved Bcl10. Cropped versions of these images are shown in Fig 1B.
(TIF)

S2 Fig RelB cleavage is impaired in activated B cells expressing MALT1 (C472A). In a separate experiment from Figs 1B and S1, purified splenic B cells from *Wt*, *Malt1* ^-/-^, and *Malt1* ^PD/PD^ mice were treated with MG-132 and ± PMA plus ionomycin for 1 h and assessed by Western blot for proteolytically cleaved RelB and Bcl10. The same samples were used for all blots shown.
(TIF)

## References

[1] Rosebeck S, Rehman AO, Lucas PC, McAllister-Lucas LM. From MALT lymphoma to the CBM signalosome: three decades of discovery. Cell Cycle. 2011; 10: 2485–2496. 21750409 10.4161/cc.10.15.16923 PMC3180188

[2] Gaide O, Favier B, Legler DF, Bonnet D, Brissoni B, Valitutti S, et al CARMA1 is a critical lipid raft-associated regulator of TCR-induced NF-kappa B activation. Nat Immunol. 2002; 3: 836–843. 12154360 10.1038/ni830

[3] Pomerantz JL, Denny EM, Baltimore D. CARD11 mediates factor-specific activation of NF-kappaB by the T cell receptor complex. EMBO J. 2002; 21: 5184–5194. 12356734 10.1093/emboj/cdf505 PMC129028

[4] Wang D, You Y, Case SM, McAllister-Lucas LM, Wang L, DiStefano PS, et al A requirement for CARMA1 in TCR-induced NF-kappa B activation. Nat Immunol. 2002; 3: 830–835. 12154356 10.1038/ni824

[5] Bertin J, Guo Y, Wang L, Srinivasula SM, Jacobson MD, Poyet JL, et al CARD9 is a novel caspase recruitment domain-containing protein that interacts with BCL10/CLAP and activates NF-kappa B. J Biol Chem. 2000; 275: 41082–41086. 11053425 10.1074/jbc.C000726200

[6] Gross O, Gewies A, Finger K, Schafer M, Sparwasser T, Peschel C, et al Card9 controls a non-TLR signalling pathway for innate anti-fungal immunity. Nature. 2006; 442: 651–656. 16862125 10.1038/nature04926

[7] Gringhuis SI, Wevers BA, Kaptein TM, van Capel TM, Theelen B, Boekhout T, et al Selective C-Rel activation via Malt1 controls anti-fungal T(H)-17 immunity by dectin-1 and dectin-2. PLoS Pathog. 2011; 7: e1001259 10.1371/journal.ppat.1001259 21283787 PMC3024268

[8] Thome M. Multifunctional roles for MALT1 in T-cell activation. Nat Rev Immunol. 2008; 8: 495–500. 10.1038/nri2338 18575460

[9] Hailfinger S, Rebeaud F, Thome M. Adapter and enzymatic functions of proteases in T-cell activation. Immunol Rev. 2009; 232: 334–347. 10.1111/j.1600-065X.2009.00830.x 19909374

[10] Oeckinghaus A, Wegener E, Welteke V, Ferch U, Arslan SC, Ruland J, et al Malt1 ubiquitination triggers NF-kappaB signaling upon T-cell activation. EMBO J. 2007; 26: 4634–4645. 17948050 10.1038/sj.emboj.7601897 PMC2080808

[11] Sun L, Deng L, Ea CK, Xia ZP, Chen ZJ. The TRAF6 ubiquitin ligase and TAK1 kinase mediate IKK activation by BCL10 and MALT1 in T lymphocytes. Mol Cell. 2004; 14: 289–301. 15125833 10.1016/s1097-2765(04)00236-9

[12] Wu CJ, Ashwell JD. NEMO recognition of ubiquitinated Bcl10 is required for T cell receptor-mediated NF-kappaB activation. Proc Natl Acad Sci U S A. 2008; 105: 3023–3028. 10.1073/pnas.0712313105 18287044 PMC2268578

[13] Zhou H, Wertz I, O'Rourke K, Ultsch M, Seshagiri S, Eby M, et al Bcl10 activates the NF-kappaB pathway through ubiquitination of NEMO. Nature. 2004; 427: 167–171. 14695475 10.1038/nature02273

[14] Ruefli-Brasse AA, French DM, Dixit VM. Regulation of NF-kappaB-dependent lymphocyte activation and development by paracaspase. Science. 2003; 302: 1581–1584. 14576442 10.1126/science.1090769

[15] Ruland J, Duncan GS, Wakeham A, Mak TW. Differential requirement for Malt1 in T and B cell antigen receptor signaling. Immunity. 2003; 19: 749–758. 14614861 10.1016/s1074-7613(03)00293-0

[16] Jost PJ, Weiss S, Ferch U, Gross O, Mak TW, Peschel C, et al Bcl10/Malt1 signaling is essential for TCR-induced NF-kappaB activation in thymocytes but dispensable for positive or negative selection. J Immunol. 2007; 178: 953–960. 17202357 10.4049/jimmunol.178.2.953

[17] Ferch U, zum Buschenfelde CM, Gewies A, Wegener E, Rauser S, Peschel C, et al MALT1 directs B cell receptor-induced canonical nuclear factor-kappaB signaling selectively to the c-Rel subunit. Nat Immunol. 2007; 8: 984–991. 17660823 10.1038/ni1493

[18] LeibundGut-Landmann S, Gross O, Robinson MJ, Osorio F, Slack EC, Tsoni SV, et al Syk- and CARD9-dependent coupling of innate immunity to the induction of T helper cells that produce interleukin 17. Nat Immunol. 2007; 8: 630–638. 17450144 10.1038/ni1460

[19] Vautier S, Sousa MG, Brown GD. C-type lectins, fungi and Th17 responses. Cytokine Growth Factor Rev. 2010; 21: 405–412. 10.1016/j.cytogfr.2010.10.001 21075040 PMC3001956

[20] Klemm S, Gutermuth J, Hultner L, Sparwasser T, Behrendt H, Peschel C, et al The Bcl10-Malt1 complex segregates Fc epsilon RI-mediated nuclear factor kappa B activation and cytokine production from mast cell degranulation. J Exp Med. 2006; 203: 337–347. 16432253 10.1084/jem.20051982 PMC2118204

[21] Klemm S, Zimmermann S, Peschel C, Mak TW, Ruland J. Bcl10 and Malt1 control lysophosphatidic acid-induced NF-kappaB activation and cytokine production. Proc Natl Acad Sci U S A. 2007; 104: 134–138. 17095601 10.1073/pnas.0608388103 PMC1765423

[22] Gross O, Grupp C, Steinberg C, Zimmermann S, Strasser D, Hannesschlager N, et al Multiple ITAM-coupled NK-cell receptors engage the Bcl10/Malt1 complex via Carma1 for NF-kappaB and MAPK activation to selectively control cytokine production. Blood. 2008; 112: 2421–2428. 10.1182/blood-2007-11-123513 18192506 PMC2532811

[23] Uren AG, O'Rourke K, Aravind LA, Pisabarro MT, Seshagiri S, Koonin EV, et al Identification of paracaspases and metacaspases: two ancient families of caspase-like proteins, one of which plays a key role in MALT lymphoma. Mol Cell. 2000; 6: 961–967. 11090634 10.1016/s1097-2765(00)00094-0

[24] Coornaert B, Baens M, Heyninck K, Bekaert T, Haegman M, Staal J, et al T cell antigen receptor stimulation induces MALT1 paracaspase-mediated cleavage of the NF-kappaB inhibitor A20. Nat Immunol. 2008; 9: 263–271. 10.1038/ni1561 18223652

[25] Rebeaud F, Hailfinger S, Posevitz-Fejfar A, Tapernoux M, Moser R, Rueda D, et al The proteolytic activity of the paracaspase MALT1 is key in T cell activation. Nat Immunol. 2008; 9: 272–281. 10.1038/ni1568 18264101

[26] Wiesmann C, Leder L, Blank J, Bernardi A, Melkko S, Decock A, et al Structural determinants of MALT1 protease activity. J Mol Biol. 2012; 419: 4–21. 10.1016/j.jmb.2012.02.018 22366302

[27] Yu JW, Jeffrey PD, Ha JY, Yang X, Shi Y. Crystal structure of the mucosa-associated lymphoid tissue lymphoma translocation 1 (MALT1) paracaspase region. Proc Natl Acad Sci U S A. 2011; 108: 21004–21009. 10.1073/pnas.1111708108 22158899 PMC3248539

[28] Duwel M, Welteke V, Oeckinghaus A, Baens M, Kloo B, Ferch U, et al A20 negatively regulates T cell receptor signaling to NF-kappaB by cleaving Malt1 ubiquitin chains. J Immunol. 2009; 182: 7718–7728. 10.4049/jimmunol.0803313 19494296

[29] Hailfinger S, Lenz G, Ngo V, Posvitz-Fejfar A, Rebeaud F, Guzzardi M, et al Essential role of MALT1 protease activity in activated B cell-like diffuse large B-cell lymphoma. Proc Natl Acad Sci U S A. 2009; 106: 19946–19951. 10.1073/pnas.0907511106 19897720 PMC2785272

[30] Uehata T, Iwasaki H, Vandenbon A, Matsushita K, Hernandez-Cuellar E, Kuniyoshi K, et al Malt1-induced cleavage of regnase-1 in CD4(+) helper T cells regulates immune activation. Cell. 2013; 153: 1036–1049. 10.1016/j.cell.2013.04.034 23706741

[31] Staal J, Driege Y, Bekaert T, Demeyer A, Muyllaert D, Van Damme P, et al T-cell receptor-induced JNK activation requires proteolytic inactivation of CYLD by MALT1. EMBO J. 2011; 30: 1742–1752. 10.1038/emboj.2011.85 21448133 PMC3101995

[32] Hailfinger S, Nogai H, Pelzer C, Jaworski M, Cabalzar K, Charton JE, et al Malt1-dependent RelB cleavage promotes canonical NF-kappaB activation in lymphocytes and lymphoma cell lines. Proc Natl Acad Sci U S A. 2011; 108: 14596–14601. 10.1073/pnas.1105020108 21873235 PMC3167514

[33] Martin F, Oliver AM, Kearney JF. Marginal zone and B1 B cells unite in the early response against T-independent blood-borne particulate antigens. Immunity. 2001; 14: 617–629. 11371363 10.1016/s1074-7613(01)00129-7

[34] Kingeter LM, Schaefer BC. Loss of protein kinase C theta, Bcl10, or Malt1 selectively impairs proliferation and NF-kappa B activation in the CD4+ T cell subset. J Immunol. 2008; 181: 6244–6254. 18941215 10.4049/jimmunol.181.9.6244 PMC2630173

[35] Kantor AB, Herzenberg LA. Origin of murine B cell lineages. Annu Rev Immunol. 1993; 11: 501–538. 8476571 10.1146/annurev.iy.11.040193.002441

[36] McAllister-Lucas LM, Baens M, Lucas PC. MALT1 protease: a new therapeutic target in B lymphoma and beyond? Clin Cancer Res. 2011; 17: 6623–6631. 10.1158/1078-0432.CCR-11-0467 21868762 PMC3207008

[37] Mc GC, Wieghofer P, Elton L, Muylaert D, Prinz M, Beyaert R, et al Paracaspase MALT1 deficiency protects mice from autoimmune-mediated demyelination. J Immunol. 2013; 190: 2896–2903. 10.4049/jimmunol.1201351 23401595

[38] Brustle A, Brenner D, Knobbe CB, Lang PA, Virtanen C, Hershenfield BM, et al The NF-kappaB regulator MALT1 determines the encephalitogenic potential of Th17 cells. J Clin Invest. 2012; 122: 4698–4709. 10.1172/JCI63528 23114599 PMC3590210

[39] Jaworski M, Marsland BJ, Gehrig J, Held W, Favre S, Luther SA, et al Malt1 protease inactivation efficiently dampens immune responses but causes spontaneous autoimmunity. EMBO J. 2014; 33: 2765–2781. 10.15252/embj.201488987 25319413 PMC4282555

[40] Gewies A, Gorka O, Bergmann H, Pechloff K, Petermann F, Jeltsch KM, et al Uncoupling Malt1 threshold function from paracaspase activity results in destructive autoimmune inflammation. Cell Rep. 2014; 9: 1292–1305. 10.1016/j.celrep.2014.10.044 25456129

