# Supplementary figures and images for: MALT1 Protease Activity Is Required for Innate and Adaptive Immune Responses
Source: PLoS One. 2015 May 12;10(5):e0127083. doi: 10.1371/journal.pone.0127083 (PMC4428694; doi:10.1371/journal.pone.0127083)

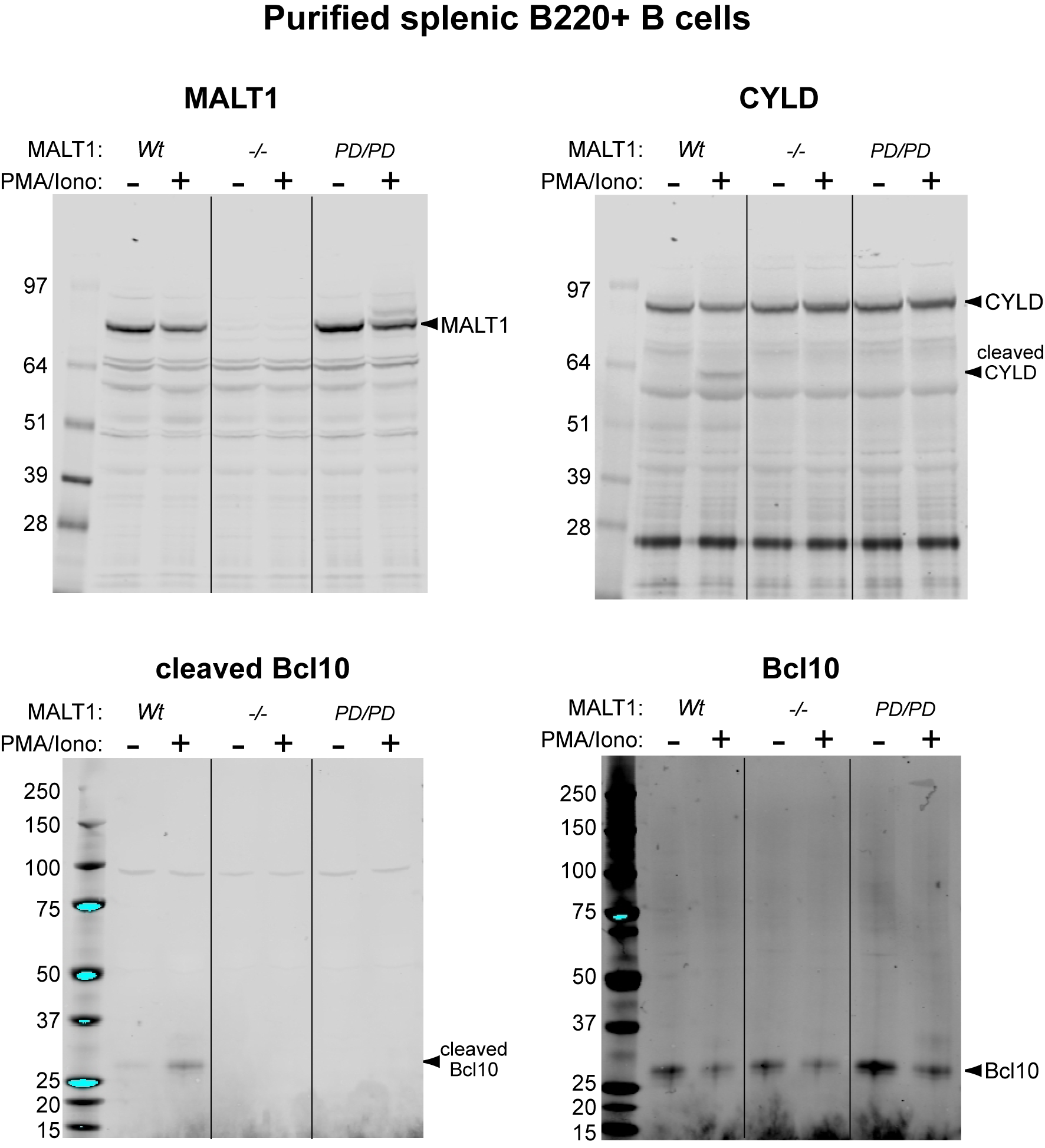

Supplement: S1 Fig — Purified total B cells from the spleens of Wt, Malt1 -/-, and Malt1 PD/PD mice were treated with (+) or without (-) PMA plus ionomycin for 1 h and assessed by Western blotting for expression of MALT1, CYLD, Bcl10, and proteolytically cleaved CYLD and Bcl10. All samples were run on the same western blot. A single blot was probed sequentially for MALT1 and CYLD. A duplicate blot with the same samples was probed for Bcl10 and cleaved Bcl10. Cropped versions of these images are shown in Fig 1B. (TIF) [file pone.0127083.s001.tif]

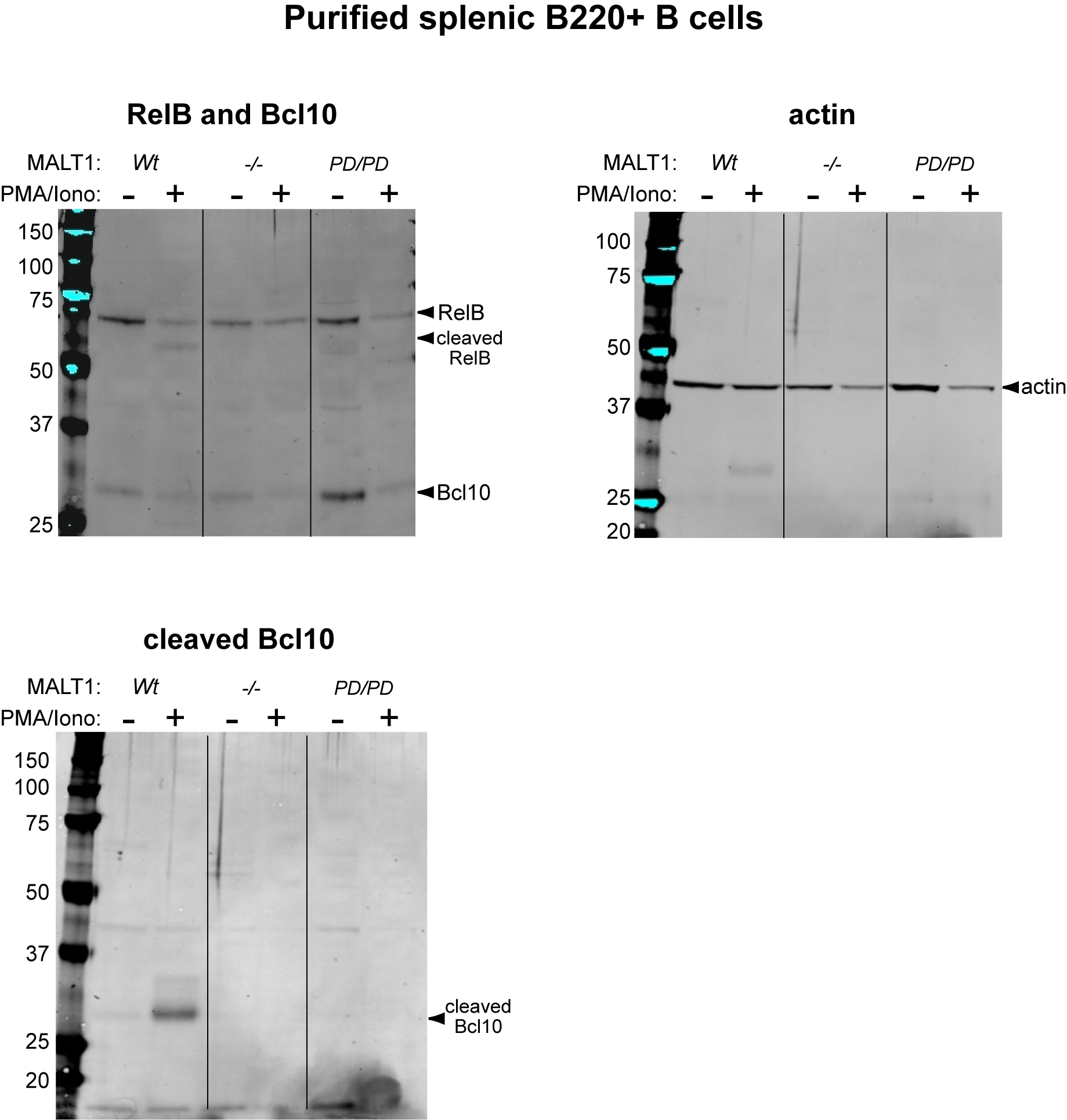

Supplement: S2 Fig — In a separate experiment from Figs 1B and S1, purified splenic B cells from Wt, Malt1 -/-, and Malt1 PD/PD mice were treated with MG-132 and ± PMA plus ionomycin for 1 h and assessed by Western blot for proteolytically cleaved RelB and Bcl10. The same samples were used for all blots shown. (TIF) [file pone.0127083.s002.tif]
